# Circulating miRNAs as Potential Biomarkers in Myasthenia Gravis: Tools for Personalized Medicine

**DOI:** 10.3389/fimmu.2020.00213

**Published:** 2020-03-04

**Authors:** Liis Sabre, Tanel Punga, Anna Rostedt Punga

**Affiliations:** ^1^Department of Neurology and Neurosurgery, University of Tartu, Tartu, Estonia; ^2^Department of Neuroscience, Clinical Neurophysiology, Uppsala University, Uppsala, Sweden; ^3^Department of Medical Biochemistry and Microbiology, Uppsala University, Uppsala, Sweden

**Keywords:** circulating microRNA, biomarker, myasthenia gravis, miR-150-5p, miR-21-5p, miR-30e-5p

## Abstract

Myasthenia gravis (MG) is an autoimmune disease caused by antibodies which attack receptors at the neuromuscular junction. One of the main difficulties in predicting the clinical course of MG is the heterogeneity of the disease, where disease progression differs greatly depending on the subgroup that the patient is classified into. MG subgroups are classified according to: age of onset [early-onset MG (EOMG; onset ≤ 50 years) *versus* late-onset MG (LOMG; onset > 50 years]; the presence of a thymoma (thymoma-associated MG); antibody subtype [acetylcholine receptor antibody seropositive (AChR+) and muscle-specific tyrosine kinase antibody seropositive (MuSK+)]; as well as clinical subtypes (ocular *versus* generalized MG). The diagnostic tests for MG, such as antibody titers, neurophysiological tests, and objective clinical fatigue score, do not necessarily reflect disease progression. Hence, there is a great need for reliable objective biomarkers in MG to follow the disease course as well as the individualized response to therapy toward personalized medicine. In this regard, circulating microRNAs (miRNAs) have emerged as promising potential biomarkers due to their accessibility in body fluids and unique profiles in different diseases, including autoimmune disorders. Several studies on circulating miRNAs in MG subtypes have revealed specific miRNA profiles in patients’ sera. In generalized AChR+ EOMG, miR-150-5p and miR-21-5p are the most elevated miRNAs, with lower levels observed upon treatment with immunosuppression and thymectomy. In AChR+ generalized LOMG, the miR-150-5p, miR-21-5p, and miR-30e-5p levels are elevated and decrease in accordance with the clinical response after immunosuppression. In ocular MG, higher levels of miR-30e-5p discriminate patients who will later generalize from those remaining ocular. In contrast, in MuSK+ MG, the levels of the let-7 miRNA family members are elevated. Studies of circulating miRNA profiles in Lrp4 or agrin antibody-seropositive MG are still lacking. This review summarizes the present knowledge of circulating miRNAs in different subgroups of MG.

## Introduction

Myasthenia gravis (MG) is a chronic autoimmune neuromuscular disorder with a prevalence of approximately 40–180 per million (1, 2). Antibodies in MG are directed against neuromuscular junction antigens; in the majority of patients, to the nicotinic acetylcholine receptor (AChR) in ∼85% and to muscle-specific tyrosine kinase (MuSK) in ∼7% of patients. More recently discovered antibody targets include the low-density lipoprotein receptor-related protein 4 (Lrp4) (3, 4) in ∼18% of AChR/MuSK antibody-seronegative patients (5) and agrin predominantly in patients with either MuSK, AChR, or Lrp4 antibodies (6, 7). MG patients suffer from fluctuating skeletal muscle fatigue and weakness. The etiology of the disease is unknown, although the thymus is considered to play a central role in the disease process as it is essential for T cell differentiation and the establishment of central tolerance (8, 9). Valid diagnostic measures for MG include antibody analysis, electrophysiological measures of impaired neuromuscular transmission, and objective clinical evaluation of skeletal muscle fatigue, such as the quantitative MG (QMG) score or MG composite (MGC) score.

One of the main difficulties in predicting the clinical course of MG is the heterogeneity of the disease, where disease progression differs greatly depending on the subgroup that the patient is classified into. The major described MG subgroups include: age of onset [early-onset MG (EOMG; onset ≤ 50 years) *versus* late-onset MG (LOMG; onset > 50 years]; the presence of a thymoma (thymoma-associated MG, TAMG); and antibody subtype [acetylcholine receptor antibody-seropositive (AChR+) *versus* muscle-specific tyrosine kinase antibody seropositive (MuSK+)] (1). In addition to the subgroups of antibody subtype, age at onset, and thymus appearance, MG in all age groups (both EOMG and LOMG) can be further subdivided according to its clinical manifestations and the muscle groups involved, mainly ocular MG (OMG) and generalized MG (GMG) (10). Besides the highly variable pattern of the initial clinical presentation of MG, skeletal muscle fatigue fluctuates over days and even hours. Antibodies to AChR, MuSK, Lrp4, or agrin also have a useful role as diagnostic biomarkers for the confirmation of MG and classifying the disease subgroup. However, their titer does not necessarily correlate with the disease severity or response to treatment (11). There is therefore a need for reliable biomarkers of disease progression as well as pharmacodynamic biomarkers that better guide therapeutic response in MG (12, 13).

According to the Food and Drug Administration (FDA) and the National Institutes of Health (NIH) Biomarker Working Group, a biomarker is defined as an easily measured indicator of a normal or abnormal physiological process or response to intervention (14). Ideally, a valid biomarker in MG should easily differentiate MG patients from healthy individuals and also be able to differentiate MG subgroups, including EOMG *versus* LOMG, AChR+ *versus* MuSK+ MG, thymoma-associated MG, as well as OMG *versus* GMG.

## Intracellular Micrornas

MicroRNAs (miRNAs) are short, endogenous non-coding RNA molecules originally discovered in roundworm *Caenorhabditis elegans* in 1993 (15, 16). With the advent of high-throughput sequencing technologies, hundreds of miRNAs have been identified in worms, flies, plants, and mammals, including humans (17). Although the number of identified human miRNAs continuously increases, only a small fraction of them has been characterized in detail. A recent study estimated that there are about 2,300 true human mature miRNAs, 1,115 of which are currently annotated in the miRbase database^1^ (V22) (18). The biosynthesis of miRNA involves cellular proteins Drosha and Dicer, which process a long primary miRNA (pri-miRNA) into ∼21–25 nucleotide double-stranded miRNA duplexes (19). Thereafter, only one RNA strand, so-called mature miRNA, is incorporated into a functional RNA-induced silencing complex (RISC) by binding to the Argonaute (Ago) proteins. Every miRNA duplex can generate two mature miRNAs: 5′-strand miRNA (known also as miRNA-5p) and 3′-strand miRNA (known also as miRNA-3p). Which of these strands is incorporated into RISC depends on the thermodynamic properties of individual miRNA duplexes (20). Remarkably, as little as 7-bp complementarities between miRNA and mRNA is enough to block targeted mRNA translation into protein (21). Considering the huge variety of mature miRNAs, it is obvious that their interactions with mRNAs regulate the key cellular processes, such as differentiation, proliferation, and apoptosis (22, 23). Not surprisingly, alterations in miRNA expression and RISC incorporation are dysregulated in many disorders, including cancer and cardiovascular and autoimmune diseases (24–26).

## Extracellular Circulating MiRNAs

### Characteristics of Circulating miRNAs

In addition to their intracellular accumulation, mature miRNAs are also detectable outside of the cells, in the extracellular space. These miRNAs, so-called circulating miRNAs, can be found in human body fluids, including blood plasma and serum, urine, saliva, semen, tears, breast milk, amniotic fluid, cerebrospinal fluid, and peritoneal and pleural fluids (27–31). The composition and the concentration of circulating miRNAs vary in different body fluids, with some distinct miRNA species dominant in specific biofluids (32).

Although miRNAs are detectable in different biofluids, the majority of circulating miRNA studies have been conducted in human serum and plasma samples due to easy access and the well-established miRNA isolation/analysis methods in this biological material. A particular feature of circulating miRNAs is that they are very stable and endure harsh treatments, such as low/high pH, high RNase concentrations, extended storage, and multiple freeze–thaw cycles (27, 30, 33). miRNAs are secreted through various types of membrane-enclosed extracellular vesicles (EVs), such as microvesicles and exosomes. The membrane encapsulation protects miRNAs from degradation and facilitates the uptake of extracellular miRNAs by the recipient cells (31, 34). The uptake of miRNAs can vary depending on the type of EV and the recipient cell origin (35). For example, tumor-derived exosomes are incorporated into organ-specific cells with the help of specific sets of exosomal integrins (36). The EVs contain specific subsets of miRNAs, which differ from the donor cell miRNA profile. An elegant study by Skog and co-workers (34) revealed that exosomal miRNA profiles differ between glioblastoma patients and healthy controls, implying that miRNAs are selectively loaded into EVs. In contrast to the well-established miRNA biogenesis, the molecular mechanisms behind miRNA loading into EVs are still poorly understood. Available studies suggest that RNA-binding proteins (e.g., Ago2 and hnRNPA2B) might provide specificity for miRNA loading into EVs (37, 38). Also, the metabolic state of a cell and its origin can influence miRNA loading into EVs (39).

### Circulating miRNAs as Potential Biomarkers

Their stability and ability to be transported in the extracellular fluids have made circulating miRNAs promising therapeutic and diagnostic tools. From the therapeutic point of view, circulating miRNAs can be considered as paracrine and endocrine signaling molecules with the ability to change gene expression on nearby and distant target cells in the body, respectively (40). Furthermore, the correlation between circulating miRNA amount and the disease outcome has put these tiny molecules in the spotlight as potential biomarkers for disease diagnosis and monitoring (39). Circulating miRNAs fulfill the requirements for a biomarker as they are specific, very stable, easily accessible in a minimally invasive manner, and their detection is cost-effective. Truly, multiple studies have shown that changes in circulating miRNA amounts can be correlated to a variety of diseases, including cancer, neurodegenerative disorders, and obesity (39). Based on that, specific changes in circulating miRNA levels can be assigned to particular diseases. For example, several studies have established circulating miRNA profiles in biofluids from obese patients. Increased levels of miR-140-5p, miR-142-3p, and miR-222, accompanied by reduced accumulations of miR-532-5p, miR-125b, miR-130b, miR-221, miR-15a, miR-423-5p, and miR-520c-3p, are reported in obese patients’ plasma (41). In addition, a liver-specific miRNA, miR-122, is found elevated in the sera of obese patients (42). These two studies not only illustrate the potential of specific circulating miRNAs as the biomarkers of obesity but also show the complexity of the circulating miRNAs in different biofluids. The use of circulating miRNAs as biomarkers is not limited to disease diagnosis and monitoring. In particular, the quantitative detection of circulating miRNAs can also be used to monitor the health of the individuals during their physical exercises and dietary regimes (43, 44).

The number of studies showing circulating miRNAs as potential biomarkers are constantly rising. However, there are still some concerns about their application as biomarkers. This involves sample collection, miRNA isolation, miRNA detection, and data analysis. The application of different sample collection and miRNA isolation protocols can lead to the under- and overrepresentation of different miRNA species. This can have serious consequences considering that the evaluation of disease status relies on a miRNA profile and not on one single miRNA (45). Quantitative reverse transcription PCR (qRT-PCR) is very often used as the standard method to evaluate miRNA expression profiles. This method is robust, easy to perform, and fast. However, normalization of the qRT-PCR data between different circulating miRNA samples is challenging due to the lack of a universal “housekeeping gene” in the EVs. Different normalization methods have been used, which, however, makes the interpretation of results puzzling and can lead to contradicting results between different studies (46). Hence, development of standardized methods and protocols, both for circulating miRNA sample processing and analysis, should reduce the present challenges and boost the practical usage of circulating miRNAs as biomarkers (47).

## Extracellular Circulating MiRNAs as Potential Biomarkers in MG Subgroups

### Acetylcholine Receptor Antibody-Seropositive Early-Onset MG

**FIGURE 1:**
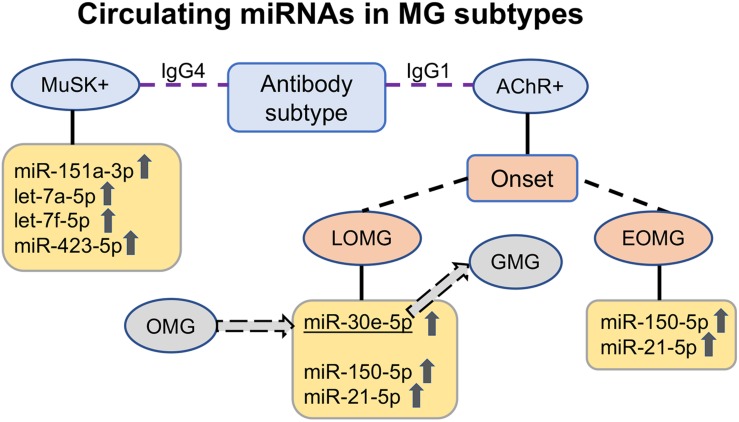
Upregulated circulating miRNAs in the serum of myasthenia gravis (MG) patients according to antibody subtype. In acetylcholine receptor antibody-seropositive (AChR +) early-onset MG (EOMG) patients, the immuno-miRs miR-150-5p and miR-21-5p are elevated; in late-onset MG (LOMG) patients, miR-30e-5p is additionally elevated. miR-30e-5p is also a potential predictor of progression from ocular to generalized MG. In muscle-specific tyrosine kinase antibody-seropositive (MuSK +) MG patients with IgG4 antibodies, another upregulated circulating miRNA profile, miR-151a-3p, let-7a-5p, let-7f-5p, and miR-423-5p, is found. *OMG* ocular MG, *GMG* generalized MG, *IgG1* immunoglobulin G subclass 1, *IgG4* immunoglobulin G subclass 4.

The first study on circulating miRNAs assessed miRNAs in the serum samples of AChR+ female generalized EOMG patients without immunosuppressive treatment (48). Extracellular miR-150-5p and miR-21-5p levels were elevated, whereas the miR-27a-3p level was reduced in MG patient sera compared to healthy controls (48). This study also indicated that miR-150-5p specifically decreased upon thymectomy, in line with clinical improvement. A follow-up study in a more heterogeneous clinical cohort of both male and female AChR+ and AChR- MG patients in 2015 also compared the miR-150-5p and miR-21-5p levels with healthy controls and patients with other autoimmune diseases, such as psoriasis and Addison’s disease (Figure 1). The levels of these two miRNAs were significantly reduced in the sera from MG patients on immunosuppressive treatment (49). This study was followed by a longitudinal study on extracellular miR-150-5p and miR-21-5p before and after thymectomy, assaying sera from 80 patients participating in the prospective international randomized trial of thymectomy in MG (MGTX Trial) (50). Longitudinal analysis of miR-150-5p and miR-21-5p indicated that the miR-150-5p levels decreased significantly 2 years after thymectomy, whereas no significant reduction was found in the prednisone group (51). The reason for the miR-150-5p levels not decreasing in the prednisone group could in part relate to the known sensitivity of miR-150 to exposure to corticosteroids, which causes levels to increase (52). These encouraging results raise the possibility of using extracellular miR-150-5p as a possible sensitive serum biomarker especially for AChR+ MG, although different effects that certain immunosuppression may have on the miR-150-5p levels should be considered (51). Intriguingly, the miR-150-5p and miR-21-5p levels decreased after physical exercise intervention in MG patients (53).

The aforementioned circulating miRNAs are not the only reported alterations in AChR+ MG patients’ biofluids. Another profiling of circulating miRNAs in different AChR+ MG patient (EOMG, LOMG, and TAMG) sera revealed that at least seven miRNAs were downregulated (miR-15b, miR-122, miR-140-3p, miR-185, miR-192, miR-20b, and miR-885-5p) compared with healthy controls (54). Nevertheless, differences in this profile of miRNAs were not found between treated and untreated MG patients (54).

### Late-Onset MG

Late-Onset MG is a specific subtype of MG where patients most often have thymus atrophy, in contrast to EOMG where thymus hyperplasia is much more frequent (55), although the majority of LOMG patients also are AChR+. LOMG is also special in that it affects relatively more male patients. In order to establish a signature of extracellular miRNA in LOMG, one separate study addressed this MG group. Compared to healthy controls, five miRNAs were found strongly elevated in LOMG patients with no immunosuppressive treatment: miR-106b-3p, miR-30e-5p, miR-223-5p, miR-140-5p, and miR-19b-3p (56). To assess the prospective influence of these miRNAs in immunosuppressive-naive generalized LOMG patients with immunosuppression, these miRNAs were longitudinally analyzed in 73 LOMG patients using sera collected for 2 years after the MG onset (56). Since 96% of these LOMG patients were AChR+, the previously found elevated miRNAs miR-21-5p and miR-150-5p (57) were also analyzed. This study found a steady decline in clinical MGC score at and after 1-year follow-up, which correlated with reduced levels of miR-150-5p, miR-21-5p, and miR-30e-5p (56; Figure 1), related to immunosuppression initiation after onset. Intriguingly, patients with generalized LOMG had higher levels of miR-150-5p and miR-21-5p than those with ocular LOMG (56). None of these miRNAs correlated with the AChR antibody levels, hence supporting the previous studies (48, 49, 51). Interestingly, the data from another cohort study on circulating miRNA in LOMG did not overlap regarding the results from the study by Nogales-Gadea (54). The discrepancies between these two studies (54, 56) could be explained by the differences in the LOMG patient cohort numbers as well as their immunosuppressive treatment status.

### Ocular MG

Ocular MG is defined as clinical MG symptoms and signs restricted to the extraocular muscles, manifesting as ptosis and/or diplopia. Retrospective studies report that up to 80% of MG patients with initially purely ocular symptoms develop secondary generalized MG (GMG), most within 2 years from disease onset (58, 59). Although there are no predictive factors for the risk of conversion from OMG to GMG, AChR+ patients are likely to be at higher risk of conversion than AChR antibody-seronegative patients (60). Since the aforementioned miRNA study in LOMG (56) revealed lower levels of some miRNAs in patients with OMG compared to GMG, a recent study aimed at determining whether circulating miRNAs could be used as potential predictors of disease generalization in MG (61). For this purpose, 83 OMG patients (82 immunosuppressive-naive) were assayed within 3 months of OMG diagnosis and at a follow-up visit. In this study, only 13 patients developed GMG. Two miRNAs were found to be significantly higher in the groups of patients who developed GMG compared to OMG: miR-30e-5p and miR-150-5p. Of these two miRNAs, miR-30e-5p had 96% sensitivity for differentiating OMG and GMG in all patients and 100% in LOMG patients (61; Figure 1). Considering that treatment with corticosteroids could modify the progression of OMG to GMG (62), and that half of the OMG patients generalize within 1 year (63), predictive biomarkers would be useful to individually tailor the immunosuppressive treatment in OMG. This could, for example, imply initiating immunosuppressive treatment at an earlier stage if the miR-30e-5p levels are higher. Hence, the study by Sabre and co-workers indicated miR-30e-5p as a potential predictor of generalization in patients with OMG symptoms (61). Another study in this field reported the downregulation of miR-20b in generalized and AChR+ OMG patients, and miR-20b expression in generalized MG was much lower than that found in OMG (64). Furthermore, the miR-20b levels increased after treatment with corticosteroids in this particular study (64).

### Muscle-Specific Tyrosine Kinase Antibody-Seropositive MG

MuSK+ MG is considered a more homogenous disease subtype that differs from AChR+ MG in pathogenesis, clinical picture, neurophysiological manifestations, and response to treatment (65). Therefore, it is not surprising that MuSK+ MG and AChR+ MG are associated with different circulating miRNA profiles. The elevated miRNAs in sera from MuSK+ MG patients instead include miR-151a-3p, let-7a-5p, let-7f-5p, and miR-423-5p (66; Figure 1). Accumulation of the aforementioned miR-150-5p or miR-21-5p, which are dysregulated in various AChR+ MG subtypes, does not differ between MuSK+ MG patients and healthy controls.

As the majority of blood samples are stored as serum, most studies have analyzed circulating miRNAs in serum. Serum and plasma contain miRNAs; however, their concentrations cannot be automatically presumed to be interchangeable (67). Recently, the miRNA profile was also analyzed in the plasma of patients with MuSK+ MG (68). Out of 179 different miRNAs, only two were distinctly different in MuSK+ MG patients; miR-210-3p and miR-324-3p were downregulated in MuSK+ MG plasma compared to healthy controls (68). None of these miRNAs have previously been reported dysregulated in immune diseases; however, miR-210-3p has been found dysregulated in several cancers (69) and miR-324-3p has been mentioned as a potential biomarker in the diagnosis of osteoporosis (70).

### Link Between Elevated Circulating miRNAs in MG and Disease Pathophysiology

Both miR-150-5p and miR-21-5p are so-called immuno-miRs and considered important T cell regulators (71). In AChR+ EOMG, the effector organ is the thymus, which is often characterized by hyperplasia as well as ectopic germinal centers consisting of infiltrating B cells (9, 72). miR-150 regulates proliferation, apoptosis, and differentiation of natural killer (NK), T, and B cells (71, 73, 74) and is a marker of lymphocyte activation (75). In addition to being elevated in the serum of EOMG patients, higher miR-150 levels are also found in other autoimmune conditions, including multiple sclerosis (MS) (76), HIV-1 infection (77), and certain cancers (78).

A recent study indicated that miR-150 expression was much higher in the thymus of AChR+ EOMG patients compared to healthy controls, in particular in the mantle zone of germinal centers containing B cells, although not directly related to the degree of thymus hyperplasia (79). In peripheral blood mononuclear cells (PBMCs), miR-150 was also downregulated in the CD4^+^ T cells of EOMG patients compared to healthy controls. The results from this study suggest that increased serum levels of miR-150-5p, which were also detected in this study, result from the released miR-150 from activated peripheral CD4^+^ T cells (79). Furthermore, miR-150 treatment of PBMCs affects the main proto-oncogene MYB, and thus, miR-150 could play a role in EOMG both at the thymic level and in the periphery by modulating the expression of target genes and peripheral cell survival (79). One hypothesis is that miR-150 could be regulated by its release into the extracellular space (80). Similar to the observations in MG, other studies demonstrate reduced miR-150-5p in PBMCs from patients with Sjogren’s syndrome, while levels are still increased in the serum and salivary glands (81, 82), in analogy with miR-150-5p in PBMCs *versus* the thymus in MG.

Another immuno-miR, miR-21-5p is expressed at higher levels in regulatory T cells (Tregs) (71) and associated with other autoimmune diseases such as systemic lupus erythematosus (SLE) and rheumatoid arthritis (24, 83). Similar to miR-150, miR-21 also plays an important role for T cells (84, 85), with higher miR-21 levels expressed in Tregs.

The third miRNA in AChR+ MG, miR-30e-5p, was somewhat contradictorily downregulated in EOMG (48) and upregulated in LOMG (56). miR-30e-5p has also been associated with SLE (86). Intriguingly, the low-density lipoprotein receptor-related protein 6 (LRP6), one of the critical co-receptors for Wnts (a family of genes that encode secretory glycoproteins), is a direct target of miR-30e (87), and thus there is a potential role for miR-30e in regulating muscle homeostasis.

The let-7 miRNA family members have been extensively studied because of their broad functional role in various cellular processes, including neuronal development and embryogenesis (88, 89). Similar to MuSK+ MG patients, the accumulation of circulating serum let-7a is observed in patients with secondary progressive MS (76). Intriguingly, let-7 miRNAs stimulate the Toll-like receptor 7 (TLR7) and thereby activate T cells (90). In addition, engagement of TLR7 in CD4^+^ T cells induces T cell unresponsiveness (91). Interestingly, at least two of the identified miRNAs, let-7a-5p and let-7f-5p, are also upregulated in PBMCs isolated from TAMG patients (92), whereas let-7f-5p is instead downregulated in the thymus of AChR+ EOMG patients (93).

As of yet, studies on circulating miRNAs in Lrp4 and agrin antibody-seropositive MG are lacking. In these MG subtypes, the antibodies as such have been suggested as biomarkers for disease (94). Further, considering the important role of agrin/Lrp4/MuSK signaling in the maintenance of the neuromuscular junction, deeper understanding of these serological subtypes is needed (94).

## Intracellular MiRNAs in MG

Studies of intracellular miRNAs in MG patients have focused mainly on PBMCs and the thymus. The first study of PBMCs revealed that 44 miRNAs are dysregulated in MG patients’ PBMCs and that let-7c expression is specifically downregulated (95). Further, miR-320a, a miRNA that modulates the expression of inflammatory cytokines, was shown to be downregulated in PBMCs from an undefined cohort of MG patients (96). Another group reported the upregulation of miR-146a in the PBMCs of AChR+ EOMG patients (97), suggesting that miR-146a might have an effect on the activation of AChR-specific B cells through the regulation of TLR4 and NF-κB. Furthermore, miR-15a expression is reduced in the PBMCs of MG patients and levels are much lower in patients with OMG compared to GMG (98). Yet another miRNA, miR-181a, is downregulated in the PBMCs of MG patients, with a negative correlation between the miR-181a level and QMG score as well as AChR antibody levels (99). Reduced miR-181c expression in the PBMCs of AChR+ MG patients also seems to correlate with elevated serum levels of the interleukins IL-7 and IL-17 (100). A recent study observed that AChR+ MG patients non-responsive to immunosuppressive treatment had lower levels of miR-323b-3p, miR-409-3p, and miR-485-3p and higher levels of miR-181d-5p and miR-340-3p in PBMCs compared to those MG patients responding to immunosuppression (101).

The first study that analyzed miRNAs in thymus cells studied TAMG and found that miR-125a-5p, which has an important role in cancer and immune processes, was significantly upregulated (92). In female EOMG patients, thymic miRNA expression analysis revealed that the most downregulated miRNAs were miR-20b-3p, miR-892-3p, and miR-7-5p (93). The most upregulated miRNAs were miR-486-5p and miR-125-5p, whereas miR-7-5p was more downregulated in the thymuses of MG patients who had high-degree thymic hyperplasia. In the MGTX Study (50), thymuses from non-thymomatous MG patients were used for miRNA and mRNA expression analysis (102). When comparing germinal center (GC)-positive samples to GC-negative ones, 38 miRNAs involved in immune response showed differences in expression (102). Regulator of G-protein signaling 13 (RGS13) is expressed in GC B cells and thymic epithelial cells. Therefore, predicted regulators of RGS13, miR-139-5p and miR-452-5p, were further analyzed and found to be downregulated (102).

## Concluding Remarks

Myasthenia gravis is a heterogeneous autoimmune disorder with several subgroups, greatly in need of easily accessible biomarkers that can aid in monitoring the disease course. The studies discussed in this review focus mainly on some of these specific subgroups of MG, including subgroups defined by antibodies (AChR+ *versus* MuSK+ MG), clinical features (GMG *versus* OMG), and age of onset (EOMG *versus* LOMG). Since there are obvious differences in the miRNA profiles between these different MG entities, further development of subgroup-specific circulating miRNA detection tests would allow for personalized medical treatment for MG patients. In the case of EOMG *versus* LOMG, they share certain upregulated miRNAs (miR-150-5p and miR-21-5p), whereas the levels of miR-30e-5p are lower in EOMG and higher in LOMG. Due to the large variations in treatment response and also in disease course over time, a crucial future need is to personalize treatment by identifying biomarkers that will predict treatment response. In this sense, circulating miRNAs could serve as indicators of disease progression for individual patients. It will be of great importance in future studies to also examine changes in miRNAs over shorter time periods to study the intra-individual and inter-individual fluctuations more closely. Differences in the response to various therapeutic agents are also subject to further studies before this will be entirely unraveled. Furthermore, all studies referred to regarding extracellular miRNAs were conducted in European populations, except for the international study on thymectomy (MGTX), and there may be important changes in the miRNA levels related to ethnicity as well as sex, which remain to be explored.

## Author Contributions

All authors contributed to manuscript drafting and revision and read and approved the submitted version.

## Conflict of Interest

The authors declare that the research was conducted in the absence of any commercial or financial relationships that could be construed as a potential conflict of interest.

## Footnotes

1 www.mirbase.org
